# Lattice 123 pattern for automated Alzheimer’s detection using EEG signal

**DOI:** 10.1007/s11571-024-10104-1

**Published:** 2024-04-03

**Authors:** Sengul Dogan, Prabal Datta Barua, Mehmet Baygin, Turker Tuncer, Ru-San Tan, Edward J. Ciaccio, Hamido Fujita, Aruna Devi, U. Rajendra Acharya

**Affiliations:** 1 Department of Digital Forensics Engineering, College of Technology, Firat University, Elazig, Turkey; 2 School of Business (Information System), University of Southern Queensland, Springfield, Australia; 3 Department of Computer Engineering, College of Engineering, Erzurum Technical University, Erzurum, Turkey; 4 Department of Cardiology, National Heart Centre Singapore, Singapore, Singapore; 5 Duke-NUS Medical School, Singapore, Singapore; 6 Department of Medicine, Columbia University Irving Medical Center, New York, NY USA; 7 Malaysia-Japan International Institute of Technology (MJIIT), Universiti Teknologi Malaysia, 54100 Kuala Lumpur, Malaysia; 8 Andalusian Research Institute in Data Science and Computational Intelligence, University of Granada, Granada, Spain; 9 Regional Research Center, Iwate Prefectural University, Iwate, Japan; 10 School of Education and Tertiary Access, University of the Sunshine Coast, Sippy Downs, Caboolture Campus, QLD Australia; 11 School of Mathematics, Physics and Computing, University of Southern Queensland, Springfield, Australia; 12 Centre for Health Research, University of Southern Queensland, Springfield, Australia

**Keywords:** Lattice123 pattern, AD detection, EEG signal classification, Feature engineering, Self-organized classification model

## Abstract

This paper presents an innovative feature engineering framework based on lattice structures for the automated identification of Alzheimer's disease (AD) using electroencephalogram (EEG) signals. Inspired by the Shannon information entropy theorem, we apply a probabilistic function to create the novel Lattice123 pattern, generating two directed graphs with minimum and maximum distance-based kernels. Using these graphs and three kernel functions (signum, upper ternary, and lower ternary), we generate six feature vectors for each input signal block to extract textural features. Multilevel discrete wavelet transform (MDWT) was used to generate low-level wavelet subbands. Our proposed model mirrors deep learning approaches, facilitating feature extraction in frequency and spatial domains at various levels. We used iterative neighborhood component analysis to select the most discriminative features from the extracted vectors. An iterative hard majority voting and a greedy algorithm were used to generate voted vectors to select the optimal channel-wise and overall results. Our proposed model yielded a classification accuracy of more than 98% and a geometric mean of more than 96%. Our proposed Lattice123 pattern, dynamic graph generation, and MDWT-based multilevel feature extraction can detect AD accurately as the proposed pattern can extract subtle changes from the EEG signal accurately. Our prototype is ready to be validated using a large and diverse database.

## Introduction

Alzheimer's disease (AD) is a neurologic disease (Ciaccio et al. 2021; Santiago and Potashkin 2021). AD patients manifest symptoms like recent memory loss (Morton et al. 2021) and, in advanced stages of the disease, the inability to perform activities of daily living (Puthusseryppady et al. 2022). Age, head trauma, environmental, and genetic factors contribute to the development of the disease (Breijyeh and Karaman 2020). AD generally affects persons aged 65 years and above, but there are also cases involving younger persons (Atri 2019). There is no definitive diagnostic test for AD (Dubois et al. 2021; Khare and Acharya 2023). Instead, doctors diagnose based on the patient's history and assessment of neurological function (Sperling et al. 2020). Blood tests and brain imaging are usually performed to exclude organic causes before confirming a final AD diagnosis (Fink et al. 2020; Wolinsky et al. 2018). While no specific treatment currently targets AD, medications can help alleviate symptoms. Additionally, physical modification of the living environment and personalized therapy may help improve the quality of life (Atri 2019).

Artificial intelligence-based automated disorder detection models have been grown since AI is one of the most effective methods to solve nondeterministic problems (Haleem et al. 2019). For instance, Acharya et al. (2019) proposed an automated model to automatically detect AD using magnetic resonance images of the brain. However, MRI is an expensive model to create an automated model. Therefore, some researchers have been used EEG signals to detect AD (Cassani et al. 2018). Our research has presented a novel handcrafted method and our model aims to generate meaningful features from EEG signals to automatically detect AD. The proposed model has been implemented on an EEG dataset and this dataset has two classes which are AD and control and proposal attained more than 98% classification performances in three experiments of the used EEG dataset.

### Literature review

In the last few years, several studies have been published on EEG-based automated diagnosis of AD and mild cognitive impairment (MCI), a lesser state impairment in cognition and activities of daily living that may lead to AD (Table 1). Several studies used deep learning-based methods (Alves et al. 2022; Bi and Wang 2019; Huggins et al. 2021; Ieracitano et al. 2019), which entail high computational complexity and costs. Some studies attained only modest classification performance (Cassani and Falk 2019; Ieracitano et al. 2019, 2020; Pirrone et al. 2022), whereas others attained high accuracy (Alves et al. 2022; Dogan et al. 2022) but on a balanced dataset.

**Table 1:** Related works on automated AD detection

Paper	Dataset	Method	Results (%)
Bi and Wang (2019)	4 healthy, 4 MCI, 4 AD	Spectral topography maps, spike convolutional deep Boltzmann machine and discriminative contractive slab	Acc: 95.04
Cassani and Falk (2019)	20 healthy, 34 AD	Spectral feature extraction, ANOVA, and SVM	Acc: 88.1 F1: 86.2
Ieracitano et al. (2019, 2020**)**	63 healthy, 63 MCI, 63 AD	Power spectral density images, custom-designed CNN	Acc: 83.3
Ieracitano et al. (2020)	63 healthy, 63 MCI, 63 AD	Continuous wavelet transform, bispectrum features, multi-layer perceptron classifier	Acc: 89.22
Huggins et al. (2021)	52 healthy, 37 MCI, 52 AD	Continuous wavelet transform, tiled topographical images, AlexNet-based CNN	Acc: 98.9
Pirrone et al. (2022)	20 healthy, 37 MCI, 48 AD	Power spectrum density, short-time Fourier transform, kNN	Acc: 86.0
Alves et al. (2022)	24 healthy, 24 AD	Pearson’s correlation, custom-designed CNN, hyperparameter optimization	Acc: 100 Pre: 100 Rec: 100
Dogan et al. (2022)	11 healthy, 12 AD	Novel primate brain pattern, iterative neighborhood component analysis, kNN	Acc: 100 Pre: 100 Rec: 100
Puri et al. (2022)	11 healthy, 12 AD	Empirical mode decomposition, Hjorth parameters using Kruskal–Wallis test, SVM	Acc: 92.90 Sen: 94.32 Spe: 94.34 Pre: 94.33 F1: 94.32
Puri et al. (2023)	11 healthy, 12 AD	Low-complexity orthogonal wavelet filter banks, SVM, wavelets	Acc: 98.60 Sen: 97.34 Spe: 99.85
Rossini et al. (2022)	16 MCI, 24 AD, 13 other dementias	Graph theory, principal components analysis, SVM	AUC: 97.00 Acc: 95.00

*Acc* accuracy, *CNN* convolutional neural network, *F1* F1 score, *kNN* k-nearest neighbor, *MCI* mild cognitive impairment, *Pre* precision, *Rec* recall, *Sen* sensitivity, *Spe* specificity, *SVM* support vector machine

### Literature gaps

The literature gaps based on Table 1 are given below:

- Most of the models developed have used conventional feature extraction and classifiers.
- Few works based on deep learning techniques have yielded high classification accuracies with high computational complexity. Training a deep model requires expensive hardware, such as graphical, tensor, or neural processing units. To enable training on simpler computer configurations, there is a need for a lightweight yet highly accurate model.

### Motivation

We have proposed a dynamic pattern-based feature extraction function, a lattice-based function, to overcome the existing literature gaps. This helps create a lightweight model that works like a deep learning model. Our presented feature engineering model is accurate with lower computational complexity than the deep learning models.

EEG depicts the spatiotemporal electrical activation of underlying brain regions recorded using a set of surface electrodes placed at standardized positions over the scalp (Friedrich et al. 2022). It has been used to study diverse neuropsychiatric conditions, including AD (Bouwman et al. 2022). However, manual interpretation of the EEG readouts from multiple electrodes (or channels) is time-intensive and requires expert knowledge (Pirrone et al. 2022), which has necessitated the development of automated methods (Pirrone et al. 2022; Puri et al. 2023; Rossini et al. 2022). We were motivated to develop an accurate and computationally lightweight model for EEG-based AD diagnosis. We adopted a handcrafted feature engineering method on a novel lattice pattern termed Lattice123. Lattices, a geometric construct common in popular science (e.g., post-quantum cryptography), have been used as directed graph pattern generators for local textural feature extraction (Cutello et al. 2007; Damewood et al. 2022; Song et al. 2022). In this work, we proposed a simple lattice pattern, Lattice123, combined with a probabilistic kernel designed to dynamically generate directed graphs for downstream textural feature extraction using binary feature generation functions akin to local binary pattern models (Ojala et al. 2002). The main contribution of this work is the innovative lattice-based dynamic feature extraction function. It searches for the optimal pattern in the EEG signal through lattice-based feature extraction. Our developed model comprises this novel lattice-based pattern and a self-organized feature engineering process. In our model, two directed graphs were generated by Lattice123 for every one-dimensional EEG input signal data block, and three binary feature generation functions were used to extract local textural features, i.e., the feature extraction function extracted 6 (= 2 × 3) feature vectors per block. Moreover, the EEG signal was decomposed using the multiple discrete wavelet transform (MDWT) (Dia et al. 2009) to partition it in the frequency domain, thereby enabling multilevel extraction of features to emulate deep modeling. Other model elements selected for their known effectiveness and computational efficiency included iterative neighborhood component analysis (INCA) feature selection (Tuncer et al. 2020b) and iterative hard majority voting (IHMV) (Dogan et al. 2021). The latter facilitated the generation of additional voted results from channel-wise outputs and the automatic selection of both channel-wise and overall best results, which rendered the model fully self-organized.

### Novelties and contributions

We have proposed a new lattice-based pattern that dynamically generated two directed graphs for extracting features using three extraction kernels. Detailed binary (AD vs. normal) channel-wise and overall classification results were presented on the multichannel EEG study dataset. The computationally lightweight and self-organized model was able to automatically generate the most suitable feature extraction graphs per the signal input and select the best channel-wise and overall voted results.

## Dataset

We used a publicly available EEG signal dataset of 59 channels to investigate facial recognition deficits for detecting AD (Mazzi et al. 2020). In this dataset, EEG signals were collected from nine participants (eight healthy individuals and one with AD) through three experiments. Participants were seated comfortably before a monitor in a dimly lit room, maintaining a fixed distance. Visual stimuli were presented on acathode ray tube (CRT)monitor using E-prime2 software, with eye movements monitored. Three experiments were conducted on different days for patients and on the same day for controls. Each trial began with a fixation cross followed by a warning tone and stimulus presentation. Participants performed a discrimination task and stimuli were presented for 300 ms.

### Experiment 1

Participants indicated whether the stimulus presented was a face, a house, or a scrambled image.

For experiments 2 and 3, participants were instructed to discriminate between upright and inverted faces.

### Experiment 2

Stimuli consisted of faces with neutral or fearful expressions.

### Experiment 3

Stimuli involved famous or unfamiliar faces.

The primary objective of these experiments was to detect amnesia or agnosia using EEG signals. We segmented each EEG signal into 15-s intervals and sampled at 250 Hz to obtain 3750 sample values. The distribution details of the dataset are shown in Table 2.

It may be noted from Table 2 that the EEG signal dataset used in this work is imbalanced.

**Table 2:** Overview of the used EEG signal dataset

No	Class	Experiment 1	Experiment 2	Experiment 3
1	Healthy	1249	1209	1376
2	AD	348	353	374
Total		1597	1562	1750

## Proposed model

**Fig. 1:**
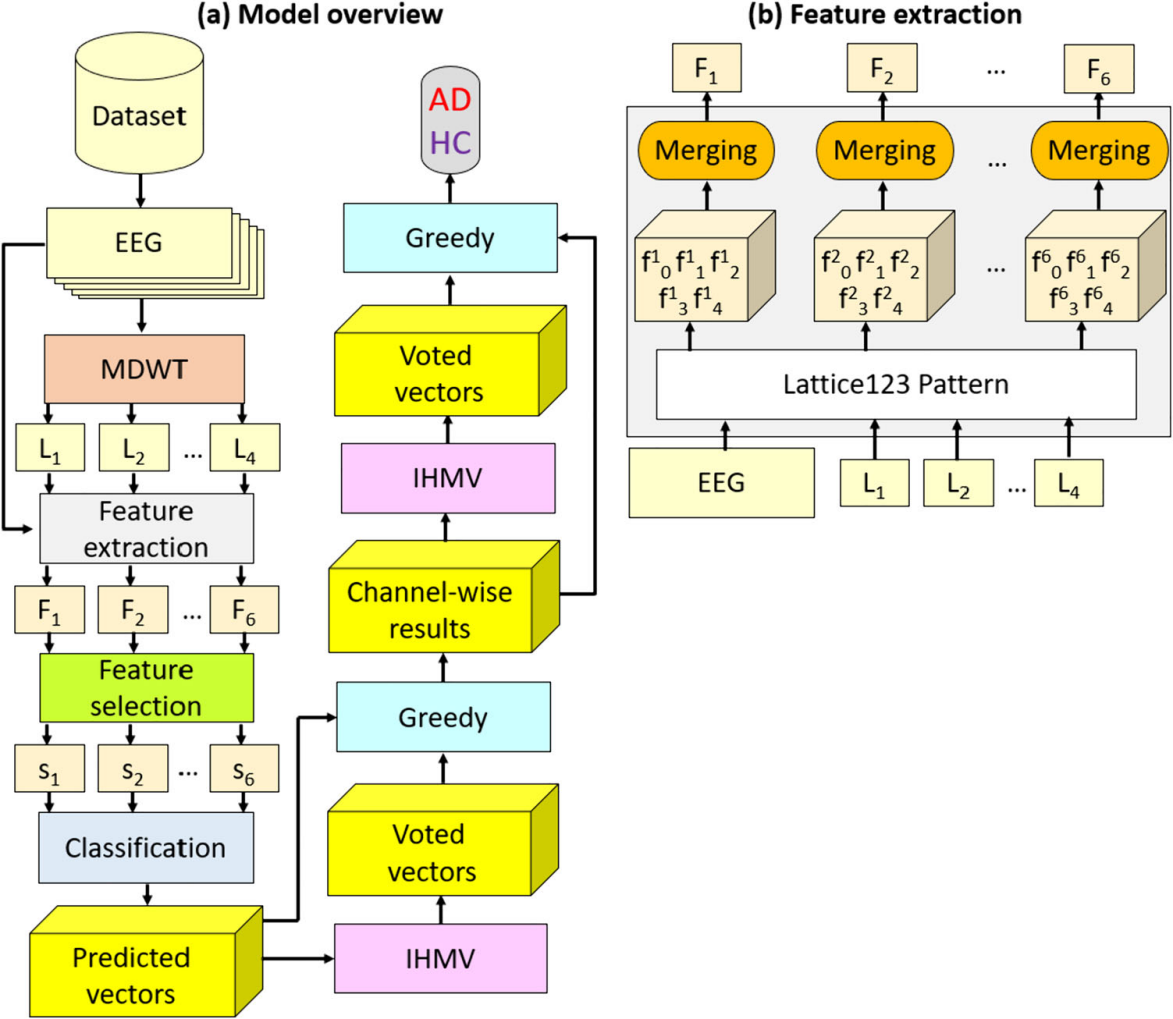
Block diagram of the proposed model: **a** model overview and **b** Lattice123-based feature extraction. In this work, we have generated two paths (maximum and minimum) by deploying the probabilistic way generation function, applying three feature extraction functions, and generating 6 (= 3 × 2) feature vectors

The self-organized AD detection model has the following layers: (1) feature extraction comprising EEG signal decomposition using MDWT (this enabled downstream multilevel feature generation, thereby mimicking deep learning) and Lattice123-based feature engineering (see section "Dataset"); (2) INCA feature selector (Tuncer et al. 2020b) to remove redundant features, thereby reducing data dimensionality; (3) a standard shallow k-nearest neighbor (kNN) classifier (Peterson 2009) to calculate channel-wise results; (4) IHMV (Dogan et al. 2021) to generate additional channel-wise voted feature vectors; (5) a greedy algorithm to calculate the best channel-wise results; and (6) IHMV plus greedy algorithm to generate additional overall voted prediction vectors and to calculate the overall best results, respectively. Our model was implemented in the MATLAB (2021a) programming environment on a computer with 16 GB memory, an Intel i7 7700 processor, and a Windows 11 operating system. The graphical clarification of the proposed Lattice123 pattern-based has been given in Fig. 1. The steps involved in each of these layers are detailed in the following subsections.

The abbreviations of this figure are as follows. AD: Alzheimer’s disease, F: concatenated extracted feature vector, f: extracted feature vector, HC: healthy control, L: low-pass filter wavelet bands, s: selected feature vector.

In this work, each EEG record contained 59 channels, each producing a spatially unique signal utilized as an input signal to the model. MDWT was applied to each signal, and four wavelet bands were generated, corresponding to four low-pass filter coefficients. The raw EEG signal and the four wavelet bands underwent Lattice123-based feature extraction to generate six feature vectors each. INCA was then applied to the generated six feature vectors to create six selected feature vectors for each signal, which were input to the kNN classifier to calculate six predicted vectors. IHMV was then applied to the predicted vectors to generate voted predicted vectors. The greedy algorithm was implemented to select the final predicted vector, representing the best channel-wise result. The 59 channel-wise final predicted vectors generated per EEG record were next input to the IHMV function to generate more voted vectors, from which the best overall binary classification result was selected using the greedy algorithm.

### Lattice123 pattern

**Fig. 2:**
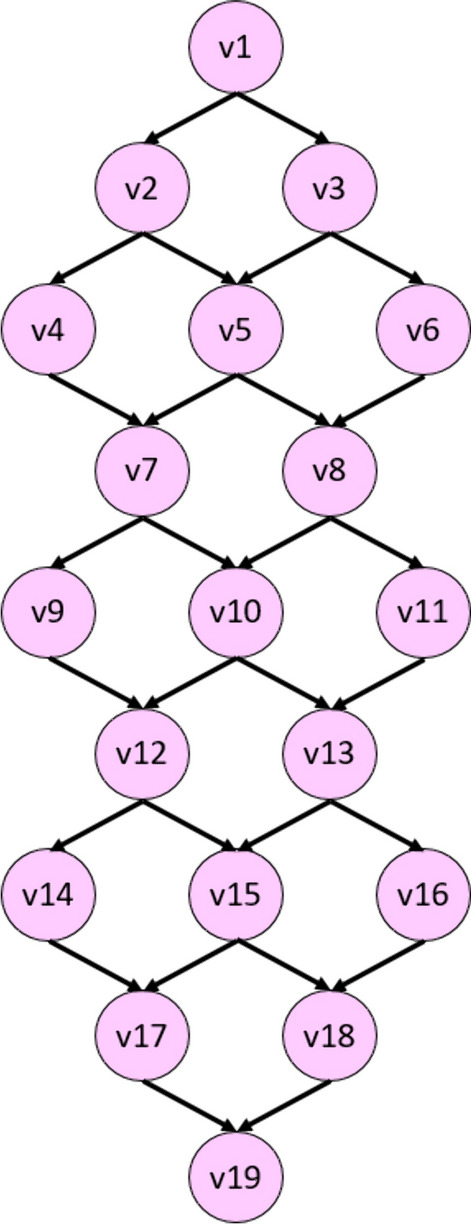
The used lattice for the graph generation. There are one (v1), two (v2 and v3), and three (v4, v5, and v6) vertexes in the top three tiers, which explains its name: Lattice123. In this research, we have used a nine-leveled Lattice123 Pattern. Therefore, we have used 19 vertexes

In graph-based feature engineering, features are generated using kernel function operations within the framework of either fixed patterns (Subasi et al. 2021; Tuncer et al. 2021a, 2021b) or adaptive patterns that are dynamically generated based on the signal input (Jiang et al. 2022; Tuncer et al. 2020a). In feature engineering, conventional feature extraction functions are employed as static patterns to generate features. However, these static patterns are limited in producing meaningful features from certain data blocks. Therefore, a dynamic feature extractor is needed to extract the hidden patterns from each block. In this research focus, we utilized the novel Lattice123 process (Fig. 2) to generate two directed graphs using a probabilistic walking path detection function.

**Fig. 3:**
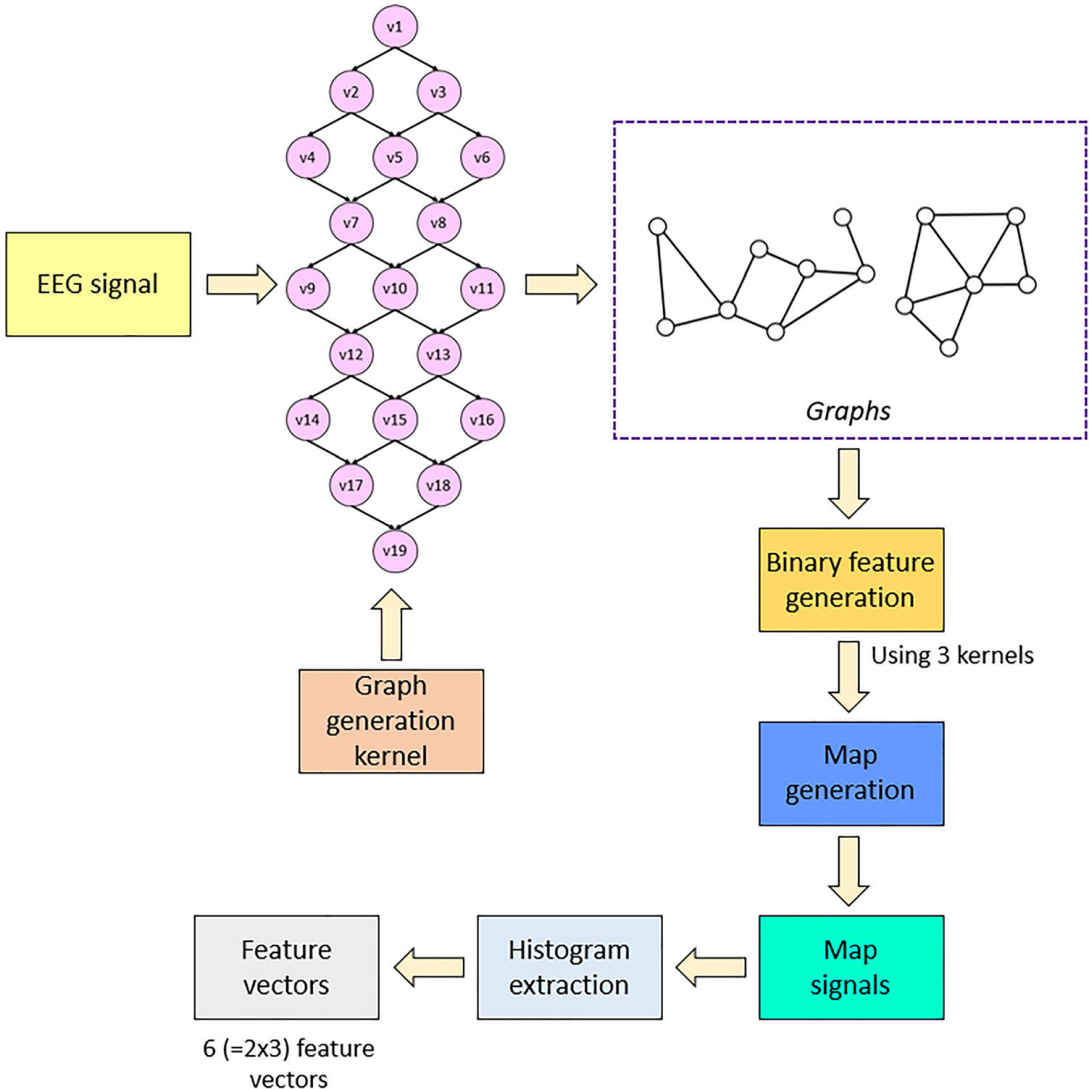
Overview of the Lattice123 pattern. In this work, we have used a one-dimensional signalsix, and we have obtained six feature vectors, and the length of each feature vector is equal to 256

The lattice used for graph generation is shown in Fig. 2. The patterns (graphs) are determined using this lattice, which comprises 19 numbered vertexes (v) and 28 directed edges (all angled downwards). First, the vertexes were populated sequentially by bit values in the input signal block. Maximum and minimum walking paths starting and ending at v1 and v19 were then calculated to generate two directed graphs for downstream (walking way) feature extraction. Histogram-based features have been extracted using the generated graphs. Therefore, the presented feature extraction model is named the Lattice123 pattern. The overview of the Lattice123 pattern is shown in Fig. 3.

The presented Lattice123 pattern is a histogram-based feature extraction algorithm, and the steps of this algorithm are given below:

1. Normalize the input signal to integer values between 1 and 100 by deploying min–max normalization.
  1 $$N = \left\lceil {\frac{{S - S_{min} }}{{S_{max} - S_{min} }}} \right\rceil \times 99 + 1$$
  where $N$ represents normalized signal; $S$, signal value; ${S}_{min}$, the minimum value of the signal; and ${S}_{max}$, the maximum value of the signal.
2. Extract the histogram of the normalized signal.
  2 $$H = \theta (N)$$
  where $H$ represents the histogram of the normalized signal; and $\theta (.)$, the histogram extraction function. In this step, we have extracted a histogram of the normalized signal.
3. Calculate the probability of each value.
  3 $$pr_{i} = \frac{{H_{i} }}{{\mathop \sum \nolimits_{i = 1}^{n} H_{i} }},i \in \{ 1,2, \ldots ,n\}$$
  where $p{r}_{i}$ represents the probability of the ith value; and $n$, the length of the signal.
4. Divide the signal into overlapping blocks of length 19.
  4 $$s\left( j \right) = S\left( {i + j - 1} \right), \;i \in \left\{ {1,2, \ldots ,n - 18} \right\},\;j \in \left\{ {1,2, \ldots ,19} \right\}$$
  5 $$v\left( j \right) = N\left( {i + j - 1} \right)$$
  where $s$ represents an overlapping block of the input signal, $S$; and $v$, the normalized overlapping block.
5. Calculate the probability matrix using probability values and relationships.
  6 $$M_{k,j} = pr_{v(j)} , k \in \left\{ {1,2, \ldots ,18} \right\}$$
  where $M$ represents the probability matrix; and ${M}_{k,j}$, the probability of the jth value, where the parent value of the jth value is the kth value.
6. Using minimization and maximization operations, create two walking paths (directed graphs) from vertex 1 to vertex 19 of the Lattice 123 pattern.
  7 $${w}_{1}^{1}=1, {w}_{1}^{2}=1,$$
  8 $${w}_{t}^{1}=argmin\left({M}_{{s}_{t-1}^{1},:}\right),t\in \{\mathrm{2,3},\dots ,8\}$$
  9 $${w}_{t}^{2}=argmax\left({M}_{{s}_{t-1}^{2},:}\right)$$
  10 $${w}_{9}^{1}=19, {w}_{9}^{2}=19$$
  where $w$ represents the walking path. In this work, we have generated two walking paths (${w}^{1}$ and ${w}^{2}$). By using a probability matrix (${M}_{{s}_{t-1}^{1},:}$) of each data block, we have generated patches and each path has nine values.
7. Extract feature vectors using the walking paths and three kernels: signum, upper ternary, and lower ternary.
  11 $$\kappa^{1} \left( {a,b} \right) = \left\{ {\begin{array}{*{20}c} {0,} & {a - b < 0} \\ {1,} & {a - b \ge 0} \\ \end{array} } \right.$$
  12 $$\kappa^{2} \left( {a,b} \right) = \left\{ {\begin{array}{*{20}c} {0,} & {a - b \le tr} \\ {1,} & {a - b > tr} \\ \end{array} } \right.$$
  13 $$\kappa^{3} \left( {a,b} \right) = \left\{ {\begin{array}{*{20}c} {0,} & {a - b \ge - tr} \\ {1,} & {a - b < - tr} \\ \end{array} } \right.$$
  where ${\kappa }^{1}(.),{\kappa }^{2}(.)$ and ${\kappa }^{3}(.)$ represent signum, upper ternary and lower ternary kernels, respectively; $a,b$, the input values of the kernels and we have used signal values as inputs; and $tr$, the threshold value for the ternary functions, which, in this model, was calculated as half the standard deviation of the signal. Six-bit groups were thus extracted using these three kernels and two walking paths.
  14 $$bit^{c} \left( t \right) = \kappa^{l} \left( {s\left( {w^{k} \left( t \right)} \right),s\left( {w^{k} \left( {t + 1} \right)} \right)} \right), \quad t \in \left\{ {1,2, \ldots ,8} \right\}k \in \left\{ {1,2} \right\},\quad l \in \left\{ {1,2,3} \right\},\quad c \in \left\{ {1,2, \ldots ,6} \right\}$$
  where $bit$ represents the binary feature array and $c$: category of the generated bit. Each $bit$ array contained eight binary features.
8. Generate feature signals (map signals) using binary-to-decimal transformation.
  15 $${m}^{c}\left(i\right)=\sum_{t=1}^{8}bi{t}^{c}\left(t\right)\times {2}^{t-1}$$
  where $m$ represents the map signal. Six map signals were generated.
9. Extract histograms of the map signals.
  16 $${H}^{c}\left(i\right)=\theta ({m}^{c})$$

Each generated histogram represents a feature vector of length 256 (= 2^8^). Six feature vectors were generated. The proposed Lattice123 pattern generates two graphs for each data block, which have been utilized as a pattern. Moreover, three kernels have been used to extract binary features for each graph. Therefore, this feature extraction method generated 6 feature vectors.

### Feature extraction

The MDWT-based decomposition of the raw input EEG signal yielded four wavelet bands. These banded signals plus the raw EEG signal were input to the Lattice123-based feature extraction model. The 11 steps that define the proposed Lattice123-based model are detailed below.

***Step 1:*** Read channel-wise signals from the EEG record of the study dataset.

***Step 2:*** Apply MDWT using Daubechies 4 (db4) mother wavelet filter function to the raw EEG signal to decompose it into four wavelet subbands corresponding to four low-pass filter coefficients.

17 $$\left[{L}_{1} {H}_{1}\right]=\vartheta (S)$$

18 $$\left[{L}_{h} {H}_{h}\right]=\vartheta \left({L}_{h-1}\right), h\in \{\mathrm{2,3},4\}$$

where $L$ represents the low-band filter; $H$, the high-band filter; and $\vartheta (.)$, the discrete wavelet transform function, $h$: number of wavelet levels.

***Step 3:*** Extract features from the raw signal and low-pass the wavelet subbands by deploying the Lattice123 pattern.

19 $$\left[{f}_{0}^{1} {f}_{0}^{2} {f}_{0}^{3} {f}_{0}^{4} {f}_{0}^{5} {f}_{0}^{6}\right]=\mathcal{L}(S)$$

20 $$\left[{f}_{t}^{1} {f}_{t}^{2} {f}_{t}^{3} {f}_{t}^{4} {f}_{t}^{5} {f}_{t}^{6}\right]=\mathcal{L}\left({L}_{t}\right), t\in \{\mathrm{1,2},\mathrm{3,4}\}$$

where $\mathcal{L}(.)$ represents the Lattice123-based feature extraction function,$S$: EEG signal, and $f$, the extracted feature vector of length 256. For instance,${f}_{0}^{1}$: the first feature vector of the raw EEG signal.

***Step 4:*** Merge the feature vectors according to type.

21 $${F}_{q}\left(j\right)={f}_{p}^{q}\left(j+\left(p\times 256\right)\right), p\in \left\{\mathrm{0,1},\dots ,4\right\},q\in \{\mathrm{1,2},\dots ,6\}$$

where $F$ represents the concatenated feature vector of length 1280 (= 256 × 5). Six concatenated feature vectors were obtained from each channel-wise input signal.

### Feature selection

We employed an iterative feature selector, an enhanced version of neighborhood component analysis (NCA), known as INCA (Tuncer et al. 2020b). It is an iterative approach used to determine the optimal number of features. It involves a series of iterations, during which additional features are systematically selected. A loss value calculation function is applied to evaluate the informativeness of the selected feature vectors in each iteration. The process continues iteratively, and the feature vector with the best-computed loss value is ultimately chosen as the final selected feature vector. The steps involved in feature selection are given below.

***Step 5:*** Apply INCA to calculate the qualified indexes of all features in each concatenated feature vector.

22 $$i{d}_{q}=\varphi ({F}_{q},y)$$

where $\varphi (.)$ represents the neighborhood component analysis feature selection function; $y$, the real output; and $id$, the qualified indexes array. The most accurate feature vector was selected using the following operations.

23 $$\begin{aligned} & fs_{q}^{r} \left( {k,j} \right) = F_{q} \left( {k,id_{q} \left( j \right)} \right),\;r \in \left\{ {1,2, \ldots ,fv - iv + 1} \right\}, \\ & \quad k \in \left\{ {1,2, \ldots ,dim} \right\}, \;j \in \left\{ {1,2, \ldots ,v} \right\},\;v \in \{ iv,iv + 1, \ldots ,fv\} . \\ \end{aligned}$$

24 $$acc_{q}^{r} = {\mathbb{C}}\left( {fs_{q}^{r} ,y} \right).$$

25 $$i{n}_{q}=argmax\left(ac{c}_{q}^{r}\right)$$

26 $${s}_{q}\left(k,z\right)={F}_{q}\left(k,i{d}_{q}\left(z\right)\right),z\in \left\{\mathrm{1,2},\dots ,i{n}_{q}+iv-1\right\}$$

where $fs$ represents the selected feature vectors; $acc$, accuracy value; ${\mathbb{C}}(.)$, the accuracy calculation function; $in$, index of most accurate feature vector; $iv$. initial value of loop; $fv$, the final value of loop; $s$, the selected final vector.

These equations describe the process of iterative feature selection using the INCA algorithm. The aim is to iteratively select and evaluate feature vectors to identify the most accurate and informative features for further processing. The loop range is set from 100 to 512, and the accuracy is obtained using the kNN classifier function.

### Calculation of channel-wise predicted vectors

The six selected feature vectors were input to a standard distance-based kNN classifier [50] to calculate the corresponding predicted vectors. The parameter settings were: k,1; distance, L1-norm; voting, no; validation and tenfold cross-validation (CV).

***Step 6:*** Classify the selected six feature vectors using the 1NN classifier (k = 1) with a tenfold CV.

27 $${p}_{q}=\delta ({s}_{q},y)$$

where $p$ represents the predicted vector; and $\delta (.)$, the kNN classifier function.

### Calculation of channel-wise voted prediction vectors

IHMV (Dogan et al. 2021) can potentially generate better results in systems that give rise to multiple results, such as our model, which produced six predicted vectors per channel. IHMV calculated qualified indexes for the predicted vectors, sorted in descending order. Then, the predicted vectors were iteratively (loop range 3 to 6) voted on by deploying the mode function, which generated additional voted vectors.

28 $$ac{c}_{q}=\Theta ({p}_{q},y)$$

29 $$id=\xi (acc)$$

30 $${vp}_{r-2}=\omega \left({p}_{id\left(j\right), }{p}_{id\left(j+1\right)}, \dots ,{p}_{id\left(r\right)}\right), r\in \{\mathrm{3,4},\dots ,np\}$$

where $\Theta (.)$ represents the accuracy calculation function; $\xi (.)$, the sorting function; $id$, are sorted indexes; $\omega (.)$, the mode function; $np$, the number of predicted vectors; and $vp$, voted prediction vector, of which four were created from the six predicted vectors generated per channel.

***Step 7:*** Apply IHMV to the six predicted vectors to create four voted prediction vectors.

### Calculation of best channel-wise result

From among the ten prediction vectors per channel (six calculated by the kNN classifier; four voted by IHMV), the greedy algorithm was applied to calculate, one at a time, the best channel-wise results for 59 channels.

***Step 8:*** Apply a greedy algorithm to select the best channel-wise result.

31 $$ac{c}_{q}=\Theta ({p}_{q},y)$$

32 $$ac{c}_{q+g}=\Theta \left({vp}_{g},y\right), g\in \{\mathrm{1,2},\mathrm{3,4}\}$$

33 $$x=max(acc)$$

where $x$ represents the index of the most accurate prediction vector and $cp$, the channel-wise prediction vector;

***Step 9:*** Repeat steps 1 to 8 until the best channel-wise results are calculated for all channels.

34 $$cp_{a} = \left\{ {\begin{array}{l} {p_{x} , x \le 6} \\ {vp_{x - 6} ,x > 6} \\ \end{array} } \right., \quad a \in \left\{ {1,2, \ldots ,nc} \right\}$$

where $nc$ represents the number of channels, i.e., 59.

### Calculation of the overall best result layer

After calculating the results of all channels, the IHMV and greedy algorithm were again applied to these results to iteratively (loop range 3 to 59) generate the overall best result for the 59-channel EEG record.

***Step 10:*** Apply IHMV to all 59 channel-wise results to generate an additional 57 (= 59–3 + 1) voted prediction vectors.

***Step 11:*** Select the most accurate predicted vector among the 116 (= 59 + 57) predicted vectors by deploying the greedy algorithm.

## Results

### Model parameters

Model parameters are summarized in Table 3.

**Table 3:** Transition table of the Lattice123-based classification model

Method	Parameters	Output
MDWT	Wavelet filter, db4; levels, n = 4; subbands, low-pass filter coefficient subbands	4 wavelet subbands
Lattice123	Block size, 19; walking path creation function, probability; generated graphs, n = 2; kernels, n = 3	The proposed feature vector generates six types of feature vectors, and each feature vector's length is 256
Feature extraction using MDWT + Lattice123	Raw EEG signal + 4 wavelet subbands used as input	6 concatenated feature vectors, each of length 1280
INCA	Loop range, 100–512; accuracy calculator, kNN	6 selected feature vectors, each of different optimal lengths
kNN	k, 1; distance, L1-norm; voting, no; validation, tenfold CV	6 predicted vectors
IHMV	Loop range, 3 to N, where N = 6 for channel-wise and N = 59 for overall result calculations; kernel, mode function	4 voted vectors were generated for each channel, and 57 were generated for overall result calculation
Greedy algorithm	Selection criteria: predicted vector with maximum accuracy	Most accurate predicted vector

### Performance metrics

Model performance for binary classification into AD versus healthy classes in the three experiments was assessed using standard metrics: accuracy and geometric mean (square root of the product of sensitivity and specificity) (Powers 2020), the latter being preferred due to the imbalanced study dataset.

### Channel-wise results

**Fig. 4:**
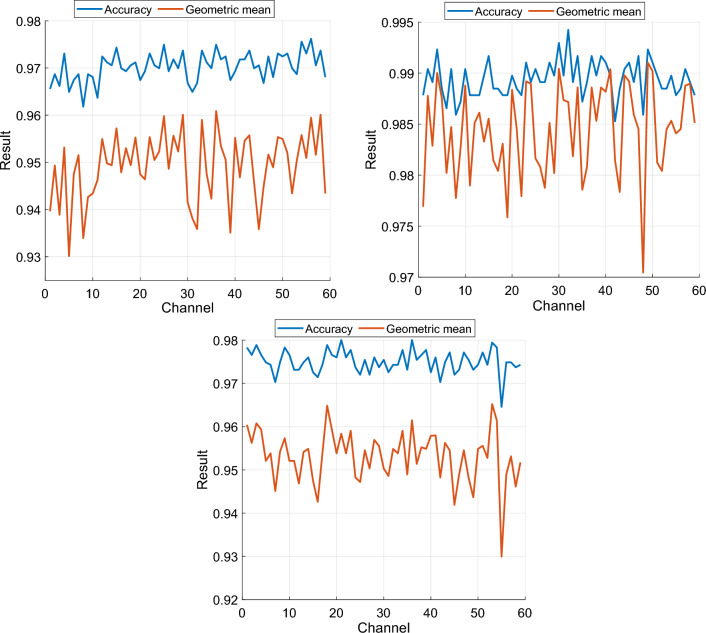
Channel-wise classification performance in the three experiments

Channel-wise results in the three experiments were excellent, with at least 96% accuracy and 93% geometric mean across all experiments (Fig. 4). For Experiments 1, 2, and 3, the best channel-wise accuracies were 97.62% (Channel 56), 99.42% (Channel 32), and 98% (Channel 21), respectively, while the best geometric means were 96.09% (Channel 36), 99.10% (Channel 49), and 96.52% (Channel 53), respectively.

**Fig. 5:**
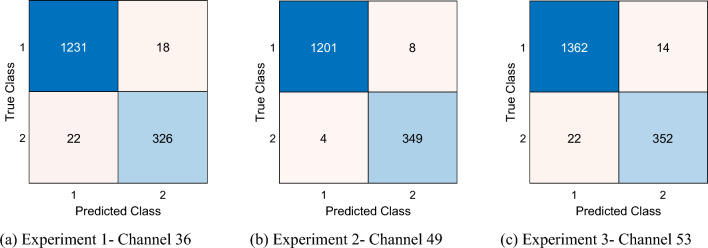
Confusion matrixes of the best channel-wise results per geometric mean. Classes 1 and 2 represent Control and AD, respectively

**Fig. 6:**
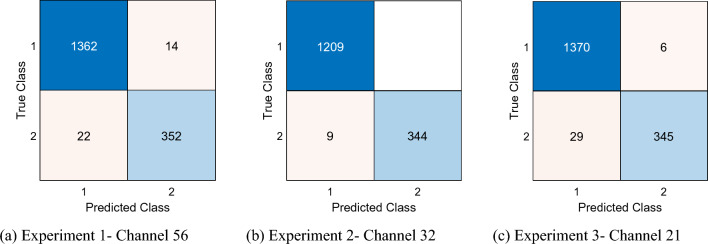
Confusion matrixes of the best channel-wise results per accuracy. Classes 1 and 2 represent Control and AD, respectively

Confusion matrixes of the best channel-wise results as ascertained by the geometric mean (Fig. 5) or accuracy criteria (Fig. 6) demonstrate low rates of misclassification, which attest to the robustness of the model.

### Overall classification results

**Fig. 7:**
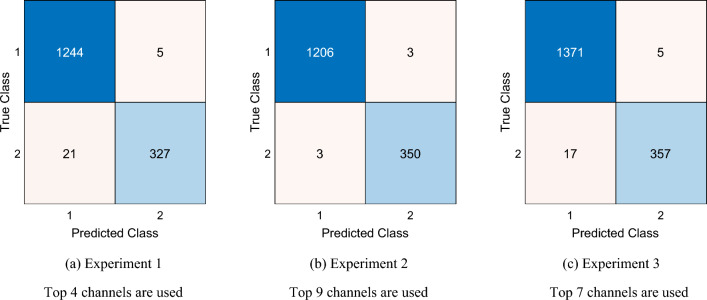
Confusion matrices of the overall best results. Classes 1 and 2 represent Control and AD classes, respectively

For Experiments 1, 2, and 3, the overall best accuracies were 98.37%, 99.62%, and 98.74%, respectively and the overall best geometric means were 96.74%, 99.45%, and 97.52%, respectively. In addition, confusion matrices of the overall best results obtained demonstrated low misclassification rates (Fig. 7).

Using Fig. 7, we have computed this model's classification accuracy, sensitivity, specificity, precision, F1-score and geometric mean. These results are presented in Table 4.

**Table 4:** Results (%) obtained using Lattice123 Pattern-based self-organized feature engineering model

Metric	Experiment 1		Experiment 2		Experiment 3	
	Class	Result	Class	Result	Class	Result
Accuracy	Control	–	Control	–	Control	–
	AD	–	AD	–	AD	–
	Overall	98.37	Overall	99.62	Overall	98.74
Sensitivity	Control	99.60	Control	99.75	Control	99.64
	AD	93.97	AD	99.15	AD	95.45
	Overall	96.79	Overall	99.45	Overall	97.55
Specificity	Control	93.97	Control	99.15	Control	95.45
	AD	99.60	AD	99.75	AD	99.64
	Overall	96.79	Overall	99.45	Overall	97.55
Precision	Control	98.34	Control	99.75	Control	98.78
	AD	98.49	AD	99.15	AD	98.62
	Overall	98.42	Overall	99.45	Overall	98.70
F1-score	Control	98.97	Control	99.75	Control	98.21
	AD	96.18	AD	99.15	AD	97.01
	Overall	97.58	Overall	99.45	Overall	98.46
Geometric mean	Control	–	Control	–	Control	–
	AD	–	AD	–	AD	–
	Overall	96.74	Overall	99.45	Overall	97.52

The results presented in Table 4 the used metrics are: accuracy, sensitivity, specificity, precision, F1-score, and geometric mean.

Our Lattice123 pattern-based self-organized feature engineering model demonstrated high performance metrics for all three experiments. In Experiment 1, the proposed model achieved 98.37% overall accuracy and this results is a high classification accuracy. Moreover, our model reached 93.97% sensitivity for AD detection and 96.74% of geometric mean was computed.

In Experiment 2 is the best accurate expirement since our model yielded 99.62% and 99.45% classification accuracy and geometric mean respectively. Moreover, our model reached 99.15% AD detection rate for this experiment.

In Experiment 3, our proposal achieved 98.64% overall classification accuracy. In this point, our model reached higher classification performance than Experiment 1 for Experiment 3.

Table 4 clearly illustrates that the presented lattice-based EEG signal classification model achieved >98% overall classification accuracies and over 93% AD detection sensitivities for all experiments. These results highlight that our proposed model has high and general (tested across three different experiments) classification performances for AD detection using EEG signals, attributable to the dynamic structure of the recommended Lattice123 feature extraction function.

### Computational complexity

The proposed handcrafted feature engineering architecture has low time complexity. Lattice123 is a dynamic pattern-based feature generator in which a probabilistic matrix was created using relations (directed edges in Fig. 1). The time burden is $O(r\times n)$, where $r$ represents the number of edges; and $n$, the length of the signal. Taking into account the signal decomposition using MDWT, the combined MDWT- and Lattice123-based multilevel feature extraction has a time burden given by $O\left(r\times n\times {\text{log}}\left(r\times n\right)\right)$. The time burden of the INCA-based feature selection is $O\left(s+lc\right)$; where $s$ represents the time complexity coefficient of the neighborhood component analysis; $l$, the number of loops; and $c$, the time complexity coefficient of the classifier—we used kNN as the classifier, which has a time complexity of $O(c)$. The computational complexity of IHMV, a basic loop-based mode function majority voting algorithm, depends on the length of the predicted vectors (number of observations) and the number of feature vectors (channels). Hence, the time complexity is $O(i\times f)$, where $i$ represents the number of iterations; and $f$, the number of observations. The time burden of the greedy algorithm is $O(a\times f)$, where $a$ represents the time complexity coefficient of the accuracy calculation. Therefore, the total time burden of our architecture is $O\left(r\times n\times {\text{log}}\left(r\times n\right)+s+lc+i\times f+a\times f\right)$, which is a linear function. Unlike deep learning architectures, there is no need for computationally intensive hyperparameter tuning.

### Comparison with the literature

We benchmarked our model against published binary AD vs. healthy classification models (Table 5). All studies used different datasets. Dogan (2022) and Alves (2022) attained 100% classification performance on balanced datasets. Using the hold-out CV strategy, Fabrizio (Vecchio et al. 2020) attained 95% accuracy on a large dataset. Cassani and Falk (2019) attained a modest 88% accuracy using a leave-one-subject-out CV. We attained over 98% accuracy in all experiments based on a small study dataset using a tenfold CV. The small dataset precluded the use of the leave-one-subject-out CV strategy. Our model attained excellent results on an imbalanced dataset, offering a good balance of performance and undemanding computational cost.

**Table 5:** Comparison of our study with published models for binary classification of Alzheimer’s disease vs. healthy control (HC)

Paper	Dataset	Method	Validation	Results (%)
Cassani and Falk (2019)	20 HC, 34 AD	Spectral feature extraction, ANOVA, SVM	LOSO CV	Acc: 88.1, F1 86.2
Vecchio et al. (2020)	120 HC, 175 AD	Exact low-resolution brain electromagnetic tomography, SVM	Hold-out CV (80:20)	Acc: 95.0, Sen: 95.0, Spe: 96.0
Alves et al. (2022)	24 HC, 24 AD	Pearson’s correlation, custom-designed CNN, hyperparameter optimization	tenfold CV	Acc: 100, Pre: 100, Rec: 100
Dogan et al. (2022)	11 HC, 12 AD	Primate brain pattern, INCA, kNN	tenfold CV	Acc: 100, Pre: 100, Rec: 100
Our model	8 HC, 1 AD	Lattice123, MDWT, INCA, kNN, IHMV, greedy algorithm	tenfold CV	Experiment 1: • Acc:98.37, GM:96.74
				Experiment 2: • Acc:99.62, GM:99.45
				Experiment 3: • Acc:98.74, GM:97.52
	Alternative dataset 11 HC, 12 AD			Acc: 100, Pre: 100, Rec: 100

## Discussion

We have presented an accurate, computationally lightweight, handcrafted lattice-based feature engineering architecture for automated AD detection using EEG signals. Inspired by the Shannon information entropy theorem (Shannon 1951), we applied a probabilistic function to a novel Lattice123 pattern to generate two directed graphs using minimum and maximum distance-based kernels (Tasci et al. 2022). Six feature vectors were produced for each input signal block using these two graphs and three kernel functions: the signum, upper ternary, and lower ternary. Moreover, MDWT-based signal decomposition gave rise to low-level wavelet subbands that enabled downstream feature extraction in the frequency and spatial domains at multiple levels, which mimicked deep models. To reduce data dimensionality, INCA selected the optimal numbers of the most discriminative features from the extracted feature vectors. Finally, the coupled IHMV and greedy algorithm were applied to generate additional voted vectors and the final selection of the best channel-wise and overall results. Our model was trained and tested on a dataset partitioned into three experiments. Excellent binary classification accuracy exceeding 98% was attained for all experiments. Moreover, the used dataset is imbalanced. Therefore, we computed other classification performance metrics as well. For instance, our model achieved over 96% geometric mean for all experiments. The computed results have been discussed below.

Across all experiments, the model consistently demonstrated exceptional performance, achieving an overall accuracy of 98.37%, 99.62%, and 98.74% in Experiments 1, 2, and 3, respectively. The overall geometric means were 96.74%, 99.45%, and 97.52% for Experiments 1, 2, and 3, respectively, further emphasizing the model's robustness. The confusion matrices obtained for the overall best results are shown in Fig. 7.

The consistent high performance across all experiments indicates that the Lattice123 Pattern-based self-organized feature engineering model effectively captures intricate patterns from the EEG signals.

Experiment 2 performed better than other experiments yielding an accuracy of 99.62%, highlighting the model’s ability to discriminate between upright and inverted faces based on EEG signals.

Hence, our presented Lattice123 Pattern-based self-organized feature engineering model is an accurate and robust automated AD detection model.

**Fig. 8:**
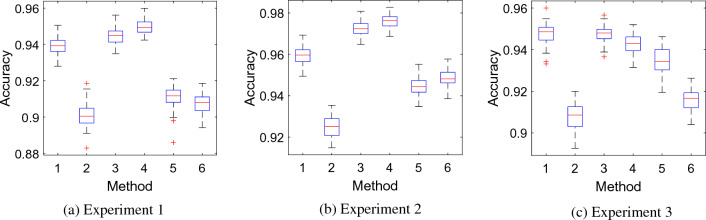
Statistical attributes of the six predicted feature vectors across all 59 channels. Herein, red lines demonstrate average classification accuracies, boxes show quartile range (Q3–Q1), and red plusses depict abnormal (extreme values per the Gaussian distribution)

To examine the relative contributions of the dynamically generated graphs and local feature extraction kernel functions to the accuracy of the Lattice123 model, we analyzed the mean accuracies of the six individual predicted feature vectors generated from every channel (Fig. 8). The combination of minimum probability + lower ternary function in Experiment 2 attained the highest accuracy.

The feature vectors are enumerated 1 to 6 based on combinations of Lattice123-generated minimum- and maximum-distance probability graphs and local textural feature extraction kernel functions: 1, minimum probability + signum function; 2, maximum probability + signum function; 3, minimum probability + upper ternary function; 4, minimum probability + lower ternary function; 5, maximum probability + upper ternary function; 6, maximum probability + lower ternary function.

**Fig. 9:**
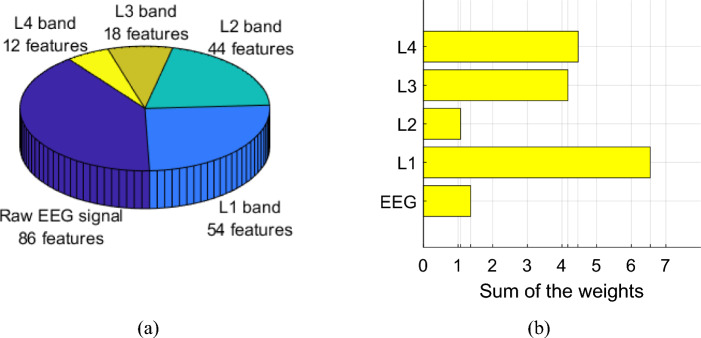
Distribution (**a**) and weight analysis (**b**) of the selected features by the type of signal input

We evaluated their feature selector indexes to examine the relative contributions of the one-dimensional raw EEG signal and the four MDWT-generated wavelet subbands to feature engineering accuracy. To standardize the comparison, we analyzed only the most accurate channel-wise performance, i.e., Channel 32 in Experiment 2 (Fig. 6), using the optimal combination of minimum-distance graph + lower ternary function (Fig. 8). Using this standardized scheme, INCA chose 214 features, which yielded a 98.37% classification accuracy. The distribution of these features across the signal input and their relative neighborhood component analysis-generated weights (Fig. 9) demonstrate that the raw EEG signal contributed the greatest number of selected features (86/214) to the channel-wise results. The most weighted signal input was the L1 wavelet subband, in which the sum of weights of its selected features was the highest at 6.55. These analyses underscore the positive effect of MDWT on feature extraction and downstream model classification performance.

**Fig. 10:**
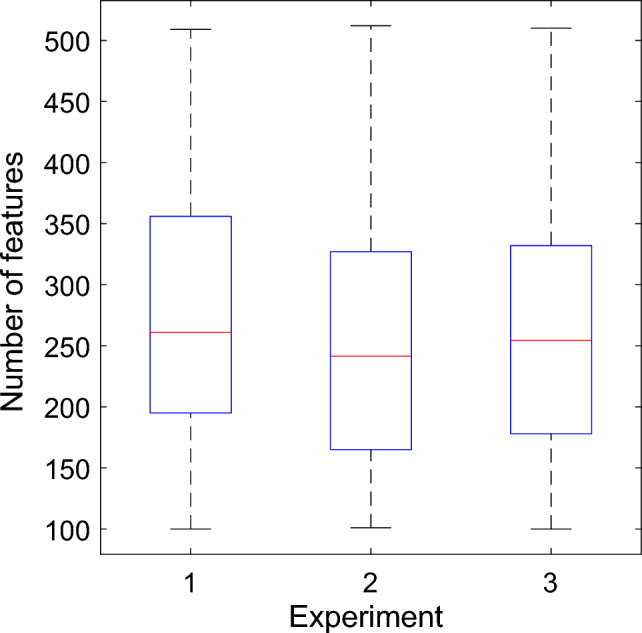
Comparison of mean lengths of selected feature vectors by experiments. In each experiment, INCA was applied 354 times to every one of the six feature vectors generated for each of the 59 channels to give rise to 354 (= 59 × 6) selected feature vectors, each of which had different optimal lengths

We also analyzed the optimal lengths of INCA-generated selected feature vectors in the three experiments. The mean lengths of the selected feature vectors were 274.02, 253.65, and 262.20 for Experiments 1, 2, and 3, respectively (Fig. 10).

Feeding the selected feature vectors to the downstream kNN classifier, the model attained (without using majority voting) accuracies of 96%, 98.27%, and 96% for Experiments 1, 2, and 3, respectively. By applying the IMHV and greedy algorithm, more accurate channel-wise results were observed, albeit on the specific best-performing single channels (see section "Overall classification results" and Figs. 3 and 5), which underscore the positive effects of majority voting. In the last layer of the model, IHMV was applied to all the best channel-wise results, and the greedy algorithm was employed to calculate the final overall best result. As a result, 98.37%, 99.62%, and 98.74% classification accuracies were attained for Experiments 1, 2, and 3, respectively, based only on limited numbers of the top 4, 9, and 7 channel-wise results. Accordingly, for the study dataset, the individual EEG channels that contributed the most toward model accuracy in all three experiments can be summarized (Table 6), the position of which may offer an element of explainability for result interpretation. For instance, EEG channels overlying the frontal region (denoted by “F” in Table 6) feature relatively prominent among valuable channels contributing to accurate AD classification.

**Table 6:** EEG channels contribute to the final overall best results in the experiments

Experiment	Channel number (spatial position of scalp electrode*)
1	56 (AF4), 54 (AF7), 25 (CP6), 36 (FT7)
2	32 (C2), 49 (Fz), 30 (C1), 4 (PO7), 15 (P4), 34 (C6), 37 (FC5), 39 (FC1), 47 (F3)
3	21 (CP1), 53 (F8), 36 (FT7), 3 (O2), 18 (TP7), 1 (O1), 9 (P7)

*Within the parentheses, capital letters A, C, F, O, P, and T refer to the anterior, central, frontal, occipital, parietal, and temporal positions of the scalp electrodes (channels) that overlie the corresponding brain regions; a small letter z refers to the mid-sagittal centerline. There is no anatomical “anterior” or "central” lobe, but the terms were used to describe the relative positions of the channels to the frontal lobe channel. Even numbers represent left-brain channels, and odd numbers represent right-brain channels

Based on the above analysis, our findings are given below:

- The proposed Lattice123 pattern produced six feature vectors per input signal block using these graphs and three kernel functions (signum, upper ternary, and lower ternary). The minimum-distance graph + lower ternary function is found to be the best combination based on our analysis.
- Mean lengths varied between 253.65 and 274.02, demonstrating diversity in selected feature vector lengths.
- Selected feature vectors coupled with the kNN classifier achieved 96%, 98.27%, and 96% accuracy for Experiments 1, 2, and 3, respectively.
- IHMV and greedy algorithm achieved the channel-wise overall accuracies of 98.37%, 99.62%, and 98.74% for Experiments 1, 2, and 3, respectively.
- Identified the EEG channels that contributed to obtaining the highest detection performance in the frontal region.

## Highlights and limitations

Highlights of the work are given below:

- We have proposed a novel Lattice123 pattern. Using a probabilistic graph generation function, directed graphs (walking paths) were dynamically generated per signal data block for downstream textural feature extraction
- The diagnostic model comprising Lattice123, multilevel feature extraction enabled by MDWT signal decomposition, INCA feature selector, kNN classifier, IHMV, and the greedy algorithm was trained and tested on an imbalanced public EEG dataset partitioned into three experiments.
- The handcrafted self-organized model attained an excellent performance level of > 98% accuracy for binary classification of AD versus healthy subjects across all three experiments, with linear computational complexity.

Limitations of our work are as follows:

- The small study dataset comprised only nine subjects, which precluded subject-wise validation.
- Default classifier settings were used. Fine-tuning operations could result in better classification performance.

## Conclusions

A novel lattice-based feature engineering model was proposed, demonstrating accuracy and computational efficiency for EEG-based AD detection. Dynamic directed graph generation by the proposed Lattice123 allowed local textural feature extraction customization specific to the input signal data block. Additionally, MDWT enabled multilevel feature generation, positively affecting model performance as assessed by the higher relative weight of decomposed wavelet subbands on feature selection. Incorporating effective information fusion methodology through IHMV and the greedy algorithm facilitated the automatic selection of the best channel-wise and overall results. The model achieved over 98% classification accuracies across all experiments in the study dataset, underscoring the advantages of the individual upstream model components. Moreover, this model is explainable since we have detected the most informative channels by using the findings of the presented Lattice123-based AD detection model. In our future work, we aim to gather larger EEG datasets to enhance our model's capabilities. We plan to incorporate extensive validation on independent datasets to address the need for validation. This validation process will enable us to accurately assess the generalizability of our proposed model across diverse scenarios. Additionally, we plan to broaden the scope of our model to include the detection of neurodegenerative disorders like, such as mild cognitive impairment (MCI), Alzheimer’s disease, Parkinson’s disease etc. Furthermore, we will explore alternative models like lattice structures to generate features and improve the classification performances. Also, we aim to provide confidence to the clinicians by implementing the explainable artificial intelligence to the proposed model (Loh et al. 2022). These enhancements will ensure that our model meets the highest standards of validation and generalizability.

## Data Availability

Not applicable.
